# Global Health Hazards From Human‐Derived Volatile Organic Compounds Amid Emission Variability

**DOI:** 10.1029/2026GH001910

**Published:** 2026-10-03

**Authors:** Annada Padhi, Suvarna Fadnavis, Mathew Koll Roxy

**Affiliations:** ^1^ Centre for Climate Change Research Indian Institute of Tropical Meteorology Ministry of Earth Sciences Pune Maharashtra India

**Keywords:** hazard quotient, hazard index, risk assessment, non‐carcinogenic effect, ECHAM‐HAMMOZ

## Abstract

Health risks from high air pollution exposure remain challenging despite pollution control strategies. Health hazards caused by particulate matter and ozone have been reported for the past three decades. Here, we report the hazard quotient for Volatile Organic Compounds (VOCs) causing non‐carcinogenic risk globally using chemistry‐climate model simulations. We report hazard quotient (HQ) for three scenarios: (a) baseline scenario, (b) continued increasing emissions leading to doubling of anthropogenic VOCs (DVOCS), and (c) mitigation scenarios where emissions are reduced to half (HalfVOCS). Interestingly, we report that long‐term ambient exposure to VOCs increases non‐carcinogenic health risk by up to 36% in the high‐emission scenario. Hazardous VOCs such as benzene, formaldehyde, hydrogen cyanide, and acetonitrile remain at levels that pose chronic risks to large populations. Benzene and formaldehyde pose the greatest hazards, with maximum HQ ranging from 1.4 to 2.0. Additionally, the DVOCS scenario increases the risk to the exposed population by 18.6%–21.5% in northern India and eastern China, and by 10.7% in West‐Central Africa. Conversely, the HalfVOCS scenario shows a decrease in the exposed population of −3 to −21%. We estimate that approximately a million people face chronic respiratory and neurological risks from current ambient VOCs levels. Our findings demonstrate that VOCs exposure constitutes a major driver of the global disease burden, necessitating immediate international emission‐reduction strategies.

## Plain Language Summary

Air pollution remains a significant global health threat, yet the risks associated with Volatile Organic Compounds (VOCs) released from vehicles, industries, and household products are poorly understood worldwide. Using chemistry‐climate model simulations, this study estimated non‐cancer health risks from VOCs exposure under three scenarios: current emissions, doubled emissions, and halved emissions. Risk was assessed using a hazard quotient, a standard measure of whether chemical exposure exceeds safe levels. The results indicate that long‐term VOCs exposure increases non‐carcinogenic health risks. Benzene and formaldehyde pose the greatest dangers, with around 1 million people currently experiencing chronic respiratory and neurological harm. If emissions are doubled, population risk increases by up to 22% in northern India, eastern China, and West‐Central Africa. Conversely, halving emissions could lower the number of people at risk. These findings highlight VOCs as a significant and underappreciated contributor to the global disease burden, making a compelling case for urgent, coordinated international strategies to reduce emissions.

## Key Points

- Long‐term VOC exposure increases global non‐carcinogenic health risk by up to 36%
- Benzene and formaldehyde dominate exposure, with hazard quotients exceeding safe limits
- Doubling VOC emissions raises exposed populations by nearly 20% in India‐China hotspots

## Graphical abstract

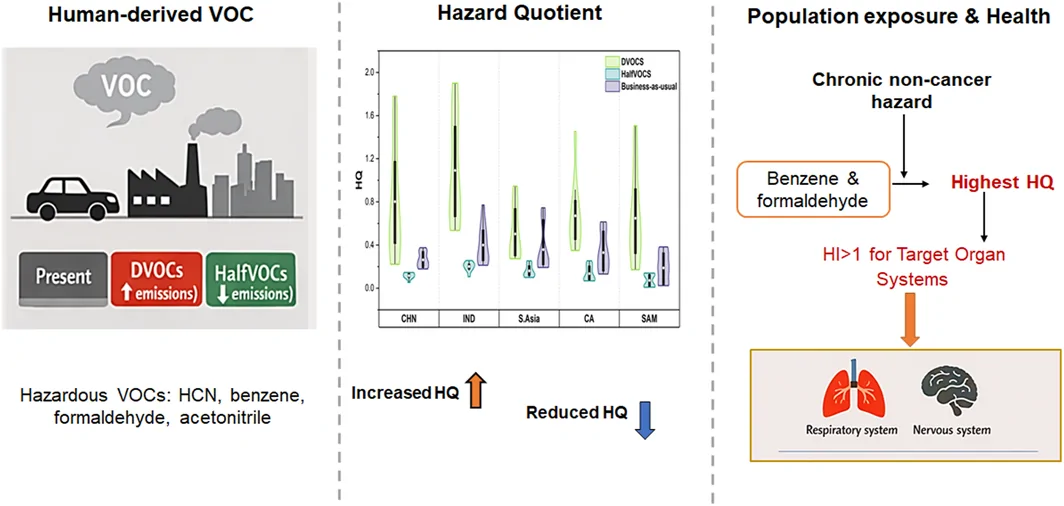

## 1 Introduction

Globally, 8.1 million people died prematurely due to air pollution in 2021 (Bhattu et al., 2024). Recent estimates indicate that air pollution is the second leading cause of global mortality, surpassing smoking and poor dietary habits (Institute for Health Metrics and Evaluation, 2025). In 2021, China and India recorded the highest numbers of air pollution‐related deaths, with each exceeding 2 million and together accounting for approximately 55% of global fatalities (Health Effects Institute, 2024). Air pollution deaths are unevenly distributed: death rates are low in high‐income nations such as the U.S., Norway, and Finland, but much higher in parts of Africa and South Asia. Pollutant emissions are also unevenly distributed worldwide, driven by rapid economic growth and high energy demand in regions such as South and East Asia and parts of Africa (Xiong et al., 2024). Consequently, their health impact is particularly severe in low‐income countries, primarily due to limited awareness, the absence of early warning systems, and restricted access to technology (Amjath‐Babu et al., 2025). As a result, the greatest effects of outdoor pollution are observed in South and East Asia, specific African regions, and rapidly developing areas in Latin America (IHME, 2025; WHO, 2021). Additionally, without any effective intervention or mitigation efforts, premature deaths in these regions are projected to rise substantially (Apte et al., 2018).

In the past, the harmful effects of criteria pollutants such as PM_2.5_, PM_10_, NO_x_, CO, and ozone on cardiorespiratory mortality and morbidity worldwide have been extensively documented (Bhattu et al., 2024 and references therein; Burnett et al., 2018; WHO, 2021; Apte et al., 2018). Detrimental health impacts caused by these pollutants are well documented in numerous studies, for example, chronic respiratory conditions like asthma, chronic obstructive pulmonary disease (COPD); and other ailments like heart attacks, leukemia, neurological issues, adverse pregnancy outcomes, kidney and liver damage, and even premature death (Kwon et al., 2018; Partha et al., 2022; Zhou et al., 2025). Unlike well‐monitored criteria pollutants, volatile organic compounds (VOCs) lack robust observational infrastructure, thereby constraining global health impact assessments (Xiong et al., 2022). Despite regulatory emphasis on PM_2_._5_, emerging evidence indicates that VOCs contribute substantially to adverse health effects yet remain underrecognised.

VOCs are emitted from natural sources, such as plants and wildfires, as well as from human activities, including vehicle emissions, industrial processes, and residential fuel use. While natural VOCs account for most of the global total, human‐derived VOCs are more harmful to health. The swift pace of industrial development and urban expansion globally, along with elevated population density, has created conditions in which anthropogenic VOCs emissions directly correlate with significant public health repercussions. Although the carcinogenic effects of species such as benzene and formaldehyde are well established (Zhou et al., 2025 and references therein), their non‐carcinogenic health impacts warrant equal scrutiny.

The chronic non‐carcinogenic health effects of VOCs affect multiple organ systems, including the respiratory, neurological, hematological, immunological, and reproductive systems. Acute exposure to VOCs has been associated with a higher risk (0.86%–1.25%) of hospitalization for respiratory conditions, including asthma and COPD (Ma et al., 2010; Qiu et al., 2021; Ran et al., 2019; Shah et al., 2013). Recent epidemiological and toxicological evidence suggests that even at concentrations below regulatory thresholds (Tables S2 and S3 in Supporting Information S1), cumulative VOCs exposures may contribute to significant morbidity and reduced quality of life (Kim et al., 2024; Kwon et al., 2018; Ran et al., 2019; Sarigiannis et al., 2011). Global anthropogenic emissions of volatile organic compounds have changed substantially over the past few decades. While some regions have achieved declining annual emissions since 2010 through control and mitigation measures (Huang et al., 2017), VOCs pollution remains high and continues to rise in China, India, other parts of Asia, and Sub‐Saharan Africa, where emissions increased significantly from 2000 to 2019 (Xiong et al., 2024). However, trends in VOCs emissions in Europe, Canada, and North America indicate that emissions are decreasing (36.4%–44%) due to mitigation policies, resulting in benefits for human health (Xiong et al., 2024).

In this study, mitigation policies refer to VOC emission‐control measures, including source‐reduction strategies, solvent‐use restrictions, low‐VOC product standards, industrial emission limits, and end‐of‐pipe control technologies (Table S4 in Supporting Information S1). Therefore, we performed three simulations: (a) the current VOCs concentrations (baseline scenario), (b) with increased emissions of anthropogenic VOCs (DVOCS), assuming no mitigation measures or regulatory efforts are in place, and (c) with reduced emissions (HalfVOCS), assuming the successful implementation of regulatory policies. The hazard quotient (HQ) and hazard index (HI) are used as metrics to assess the health risks posed by VOCs (OEHHA, 2003). Hazard quotients for VOCs frequently exceed 1 in polluted areas, indicating non‐cancer risks from long‐term exposure. Several studies across Asia report HQ > 1 for benzene, formaldehyde, and acrolein (Liu et al., 2023 and references therein). While acknowledging that humans spend most of their time indoors where VOCs from anthropogenic sources often reach high concentrations, this study deliberately concentrates on outdoor/ambient VOCs. This focus arises from the unique challenges of global outdoor VOCs monitoring, as comprehensive measurements remain rare and are largely limited to a few stations in developed regions. By examining emission variability and associated health hazards using available ambient data sets, our work fills an important gap in understanding large‐scale, population‐level risks, complementing indoor exposure studies and guiding broader air quality policies.

The results from this study are critical since VOCs‐related health impacts remain challenging due to limited ground‐based and satellite data, particularly for toxic compounds such as benzene. Sparse monitoring sites in major regions and the indirect nature of satellite observations hinder accurate global assessments and modeling of VOCs exposure and health risks. Hence, this study seeks to address the research gap on the chronic non‐cancer hazards posed by VOCs at a global scale, across varying anthropogenic emissions scenarios, using chemistry‐climate model simulations.

## 2 Methodology

### 2.1 Model Setup and Experiment

In this study, we utilize the state‐of‐the‐art chemistry‐climate model, ECHAM6.3‐HAM2.3‐MOZ1.0 (Schultz et al., 2018), to examine the global long‐term effects of hazardous volatile organic compounds (HazVOCs) at the surface. The ECHAM‐HAMMOZ model has been extensively used to simulate global tropospheric and stratospheric atmospheric composition (Fadnavis et al., 2023, 2025). It comprises the Hamburg Aerosol module (HAM), the chemistry module from MOZART‐4 (MOZ) (Emmons et al., 2010), and the general circulation model ECHAM. The comprehensive chemistry mechanisms used in the current version are an expansion and upgrade of the MOZART‐4 model for the oxidation of terpenes and aromatics. MOZ employs the same chemical pre‐processor as CAM‐Chem (Lamarque et al., 2010) and WACCM (Kinnison et al., 2007) to generate a FORTRAN code containing the chemical solver for chemical mechanisms. The MOZ sub‐model includes several primary non‐methane hydrocarbons (NMHCs) and related oxygenated organic compounds. The model emission inventory used for model simulations is the Atmospheric Chemistry and Climate Model Intercomparison Project (ACCMIP) (Lamarque et al., 2010; van Vuuren et al., 2011).

Details of model parameterizations and validation are described by Fadnavis, Müller, et al., 2021, Fadnavis, Sabin, et al., 2021; Fadnavis et al., 2022, 2023. Additional information about the model simulation used in the study is provided in Table S1 of Supporting Information S1. In this study, out of the total 21 VOCs in the model output, we analyzed the non‐carcinogenic risks of 11 HazVOCs (for which reference exposure limits are available), namely formaldehyde, acetaldehyde, acetonitrile, benzene, hydrogen cyanide, toluene, xylenes, propylene, methanol, methyl ethyl ketone, and carbon tetrachloride at 5‐year intervals from 2001 to 2019 considered for baseline scenario (Table S2 in Supporting Information S1).

We performed two emission sensitivity experiments: (a) increasing anthropogenic VOCs emission levels globally by 100%, hereafter referred to as DVOCS; (b) decreasing the emission levels to Half, referred to as HalfVOCS. In both cases, the emissions were perturbed relative to a scenario hereafter referred to as the baseline scenario. In each experiment, we specified the monthly‐varying AMIP2 sea surface temperature and sea ice fields representative of 2001–2019 as the lower boundary conditions. We compared the baseline, DVOCS, and HalfVOCS simulations to assess the spatial distribution patterns and hazard risk of HazVOCs.

Following the approach by Xiong et al. (2024), before conducting the risk assessment, we calculated the population‐weighted concentrations of each VOCs, as shown in Equation 1

(1) $${\text{PWC}}_{i,j,k}=\frac{\sum {\text{POP}}_{i,j\;}\times {\text{Conc}}_{i,j,k}}{\sum {\text{POP}}_{i,j}}.$$

Where PWC_*i*, *j*, *k*_ refers to the population‐weighted annual mean concentration of species (*k*) in the unit of μgm^−3^ within grid box (*i*, *j*). Data on global population counts were obtained from the NASA Socioeconomic Data and Application Centre for the Gridded Population of the World, version 4 (GPWv4), with a horizontal resolution of 1°. Conc_*i*, *j*, *k*_ represents the annual mean concentration of species (*k*) μgm^−3^ within the horizontal grid box (*i*, *j*).

### 2.2 Chronic Non‐Cancer‐Associated Risk

For non‐cancer hazard assessment, chronic inhalation reference exposure limits (RELs) are used as indicators of potential non‐cancer effects. REL is defined as the concentration at or below which no adverse non‐cancer health effects are anticipated following long‐term exposure (OEHHA, 2003). To estimate the chronic non‐cancer‐associated risk due to inhalation exposure to 11 HazVOCs, the hazard quotient (HQ) for each VOCs species and the hazard index (HI) for a target organ system were calculated using the following equations‐

(2) $$\text{Chronic}\;HQ_{i,j,k}=\text{PW}C_{i,j,k}/\text{RE}L_{k}$$

(3) $${\text{HI}}_{i,j,m=}\sum_{m=1}^{n}{\text{HQ}}_{i,j,k}.$$

Where Chronic HQ_*i*, *j*, *k*_ denotes the chronic hazard quotient of VOCs species (*k*) within each grid box (*i*, *j*); PWC_*i*, *j*, *k*_ refers to the population‐weighted annual mean concentration of species (*k*) in the unit of μg m^−3^ within grid box (*i*, *j*); REL_*k*_ is the reference exposure level (μg m^−3^) for the particular species (*k*). Equation 2 describes the equation used to compute the hazard index (HI) for the target organ system (e.g., respiratory, cardiovascular, nervous, etc.). This index is calculated by summing up the HQ values for each hazardous VOCs associated with the same target organ system (Table S3 in Supporting Information S1). HI_*i*, *j*, *m*_ denotes the hazard index for target organ (*m*) in the horizontal grid box (*i*, *j*). We recognize that PM_2.5_ and hazardous VOCs also pose a significant inhalation cancer risk at ambient exposure levels. This study focuses on non‐carcinogenic risks from human‐derived ambient VOCs due to emission variability, enabling evaluation of endpoints such as respiratory, neurological, and cardiovascular effects using hazard quotient methods. Carcinogenic risks are acknowledged but deferred to future research with expanded data and modeling.

### 2.3 Model Validation

To evaluate model performance of surface concentrations of VOCs, we compared model‐simulated formaldehyde concentrations with monthly averaged data from OMI/MLS satellite retrievals for 2005–2019 (Figure 1). Figures 1a and 1b show monthly‐averaged tropospheric HCHO columns compared with OMI/MLS (2005–2019) retrievals (Figures 1a and 1b). The model reproduces the observed seasonal cycle in the Northern Hemisphere, though with an overestimation, most pronounced in tropical regions by 0.6–2.9 (0.5–1.7) × 10^15^ molecules cm^−2^ during the NH from May to October (Fadnavis et al., 2025). Quantitatively, significant overestimations (in the model vs. satellite observations, *p* < 0.05) in the mid‐latitudes (20°N/S–40°N/S) ranged from 0.3 to 0.5 × 10^15^ molecules cm^2^ in the Northern Hemisphere (Figure 1a), and 0.4–1.0 × 10^15^ and 0.5–1.4 × 10^15^ molecules cm^2^ in the Southern Hemisphere (Figure 1b).

**Figure 1:**
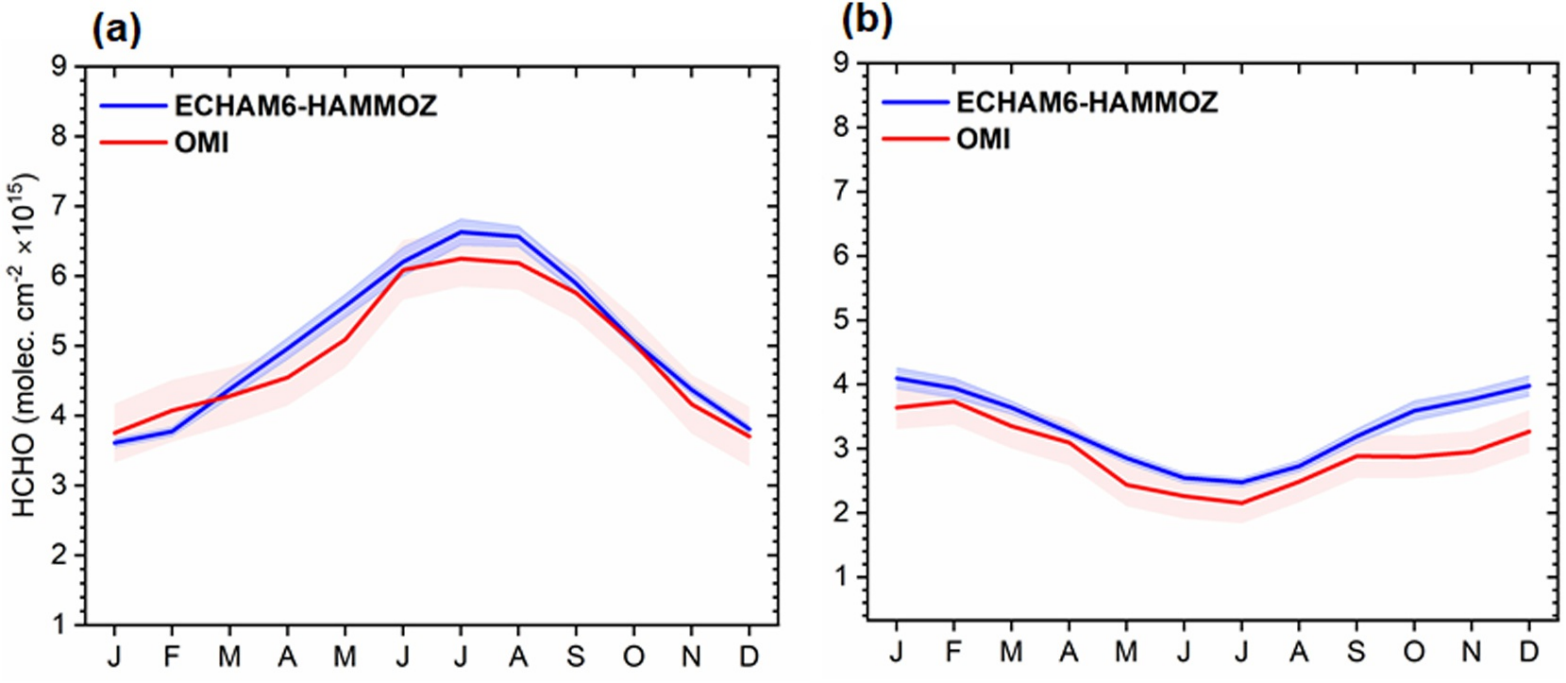
(a) Time‐series of monthly mean tropospheric column formaldehyde (HCHO) (molecules/cm^2^) averaged for 20° wide latitude bins (20°N–40°N) from ECHAM6–HAMMOZ baseline simulations (blue) and HCHO from OMI/MLS for the period January 2005 to December 2019 (b) same as (a) for (20°S–40°S) The shaded area in the figures represent standard error of the monthly mean.

For short‐lived, photochemically active species like HCHO, continuously produced and removed by OH radical chemistry, observational limitations are minor (see Figures 2m–2x in Fadnavis et al., 2025). Our study builds on this validated framework, using the same model and evaluation strategy as (Fadnavis et al., 2025) to ensure robustness in analyzing the spatial and seasonal patterns of anthropogenic VOCs. The estimated 1sigma precision for the OMI/MLS monthly mean grided tropospheric column ozone was about 1.3 DU.The adjustments for drift calibration and other issues affecting OMI/MLS tropospheric column ozone are further discussed in (Fadnavis et al., 2025 and references therein).

Ground‐based monitoring networks for VOCs (especially BTEX compounds: benzene, toluene, ethyl benzene and xylene) are primarily located in the United States, Europe, and Canada. However, the regions focused on in this study, namely, India, South Asia (Indonesia and Malaysia), Central Africa, and China, lack extensive ground monitoring infrastructure, and their data are often not publicly available. As a result, we rely on satellite data, which provides continuous spatial coverage across these regions and offers long‐term records suitable for multi‐year studies.

## 3 Results and Discussion

### 3.1 Global Distribution of Hazardous VOCs

This section presents the spatial distribution of mean annual emissions of 11 selected hazardous volatile organic compounds (HazVOCs) globally for the period 2001–2019 (in kg m^2^ s^−1^ × 10^−12^). The emissions exhibit spatial heterogeneity, with the highest emissions in densely populated and industrialized regions (Figure 2a). Peak emission intensities exceed 200 kg m^2^ s^−1^ × 10^−12^ in equatorial Africa (particularly the central and western parts), Southeast Asia, East Asia, India and parts of South America. This is due to emissions from biomass burning, agricultural activities, and industrial sources in these tropical regions (Duan et al., 2023; Gidden et al., 2019; Huang et al., 2017, 2023; Klimont et al., 2017). Moderate to low emissions (40–120) kg m^2^ s^−1^ × 10^−12^ are evident across eastern North America, Europe, including Canada, where major control policies have been implemented since 2010 (Huang et al., 2023). This spatial pattern underscores the significant contribution of both anthropogenic activities and biogenic sources to the global HazVOCs burden, with tropical regions emerging as major emission hotspots during the study period.

**Figure 2:**
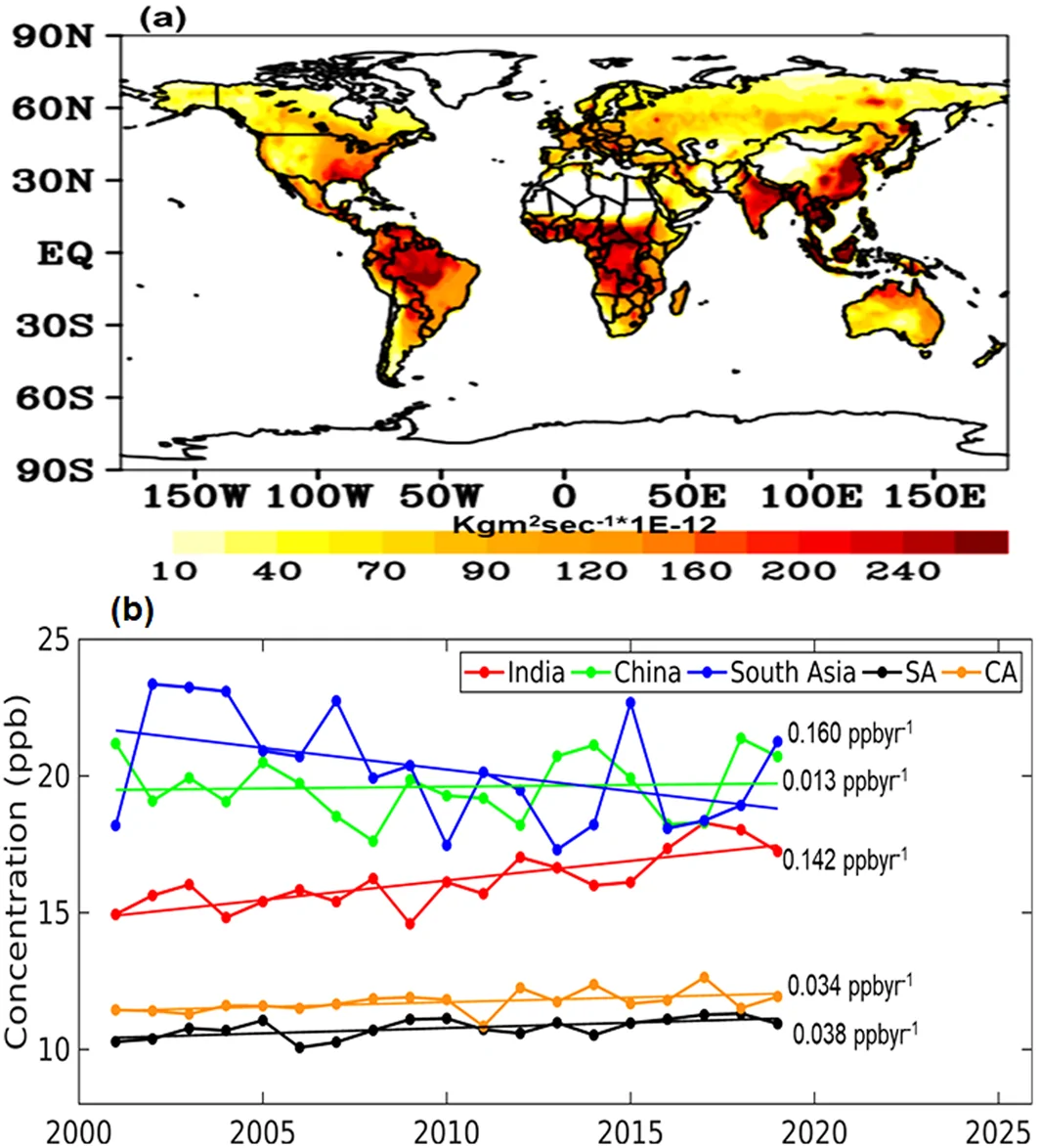
(a) Annual mean emission of 11 hazardous VOCs from 2001 to 2019 from the model and (b) Interannual variation of total hazardous VOCs in high emission regions‐ India, China, South Asia, South America and Central Africa.

Further, we analyzed annual de‐seasonalized trends in the concentration of HazVOCs in the major high‐emission regions shown in Figure 2b. Increasing annual trends are observed in regions such as Central Africa (0.034 ppb yr^−1^, *p*‐value = 0.0015), South Asia (0.160 ppb yr^−1^, *p*‐value = 0.04), India (0.142 ppb yr^−1^, *p*‐value = 0.0003), South America (0.038 ppb yr^−1^, *p*‐value = 0.0013), and China (0.013 ppb yr^−1^, *p*‐value = 0.05). A negative trend is observed in North America and Western Europe (0.001 ppb yr^−1^) and is therefore excluded from this study. The interannual trends in HazVOCs concentrations are consistent with recent studies (Fadnavis et al., 2025; Mozaffar & Zhang, 2020).

Figure 3 shows the global distribution of population‐weighted concentrations of total hazardous volatile organic compounds (HazVOCs) in 2019, expressed in μg m^−3^. The population‐weighting approach identifies regions where high pollutant concentrations coincide with densely populated areas. Globally, the average annual concentration of HazVOCs declined from 8.2 μg m^−3^ in 2001 to 7.6 μg m^−3^ in 2019. Notably, despite a global decline in mean HazVOCs concentrations, annual trends are increasing in East Asia, Africa, and other regions, as shown previously in Figure 2b.

**Figure 3:**
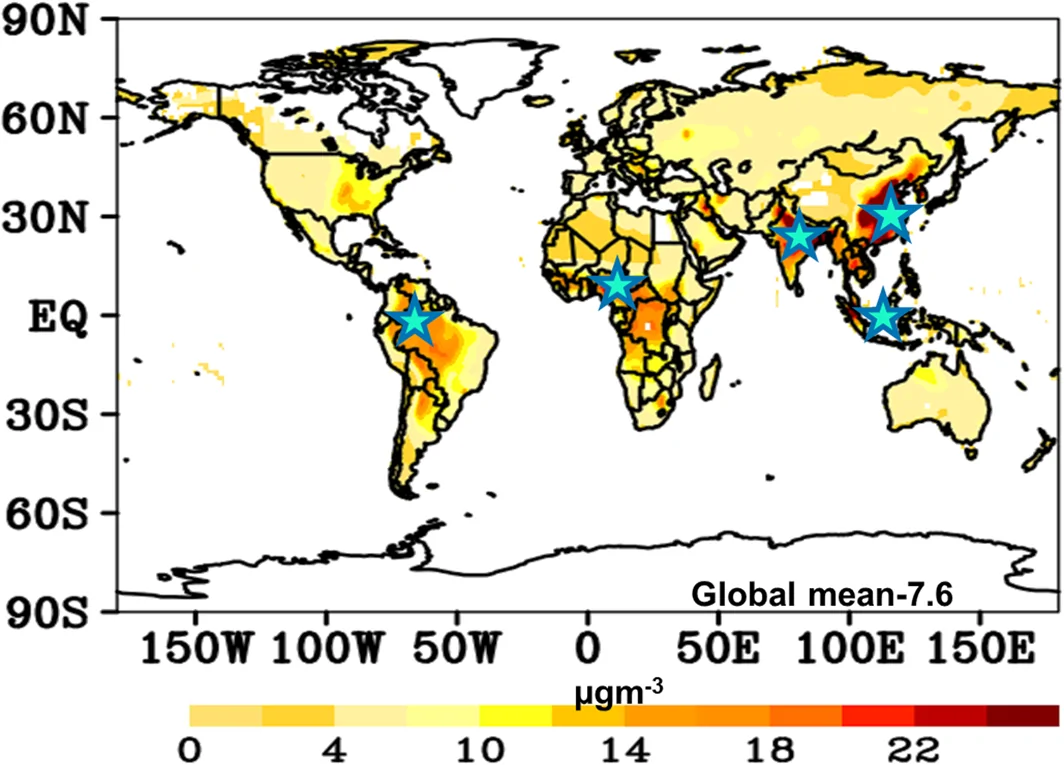
Global distribution of population‐weighted concentration of total hazardous VOCs in 2019, (*) shows regions considered in the study.

The highest population‐weighted concentrations, exceeding 22 μg m^−3^, are seen in eastern and southern Asia, particularly over the Indian subcontinent and parts of China, reflecting the convergence of substantial anthropogenic emissions with high population densities in these rapidly developing regions. Moderate concentrations (10–18 μg m^−3^) are evident across central Africa, parts of South America, and Southeast Asia, indicating considerable population exposure in developing regions. The spatial pattern clearly shows that Eastern China [20°N–38°N, 106°E–122°E] referred as CHN, Northern India mostly constituting the Indo‐Gangetic Plain (IGP) region [18°N–36°N, 73°E–85°E] referred as IND, South Asia [98°E–120°E, 10°S–6°N] referred as S.Asia, western central Africa [12°S–10°N, 2°W–31°E] referred as CA, and northern part of South America [10°S–4°N, 70°W–50°W] refereed as SAM as critical regions of concern for public health impacts from HazVOCs exposure.

### 3.2 Chronic Non‐Cancer Risk Assessment

#### 3.2.1 Hazard Quotient Spatial Distribution

The spatial patterns of hazard quotients (HQs) for hydrogen cyanide (HCN), acetonitrile (CH_3_CN), formaldehyde (CH_2_O), and benzene reveal significant regional variations in potential non‐carcinogenic health risks (Figure 4). HQ values exceeding unity indicate that exposure concentrations exceed the reference dose or Reference Exposure Level (REL) established by USEPA, suggesting potential adverse health effects. For other HazVOCs, such as xylene, toluene, methyl alcohol, acetaldehyde, carbon tetrachloride, and methyl ethyl ketone (MEK), the HQ values were significantly below the safe level of ≤1 (Figure 4a), indicating that exposure to these HazVOCs would not result in adverse effects in most regions.

**Figure 4:**
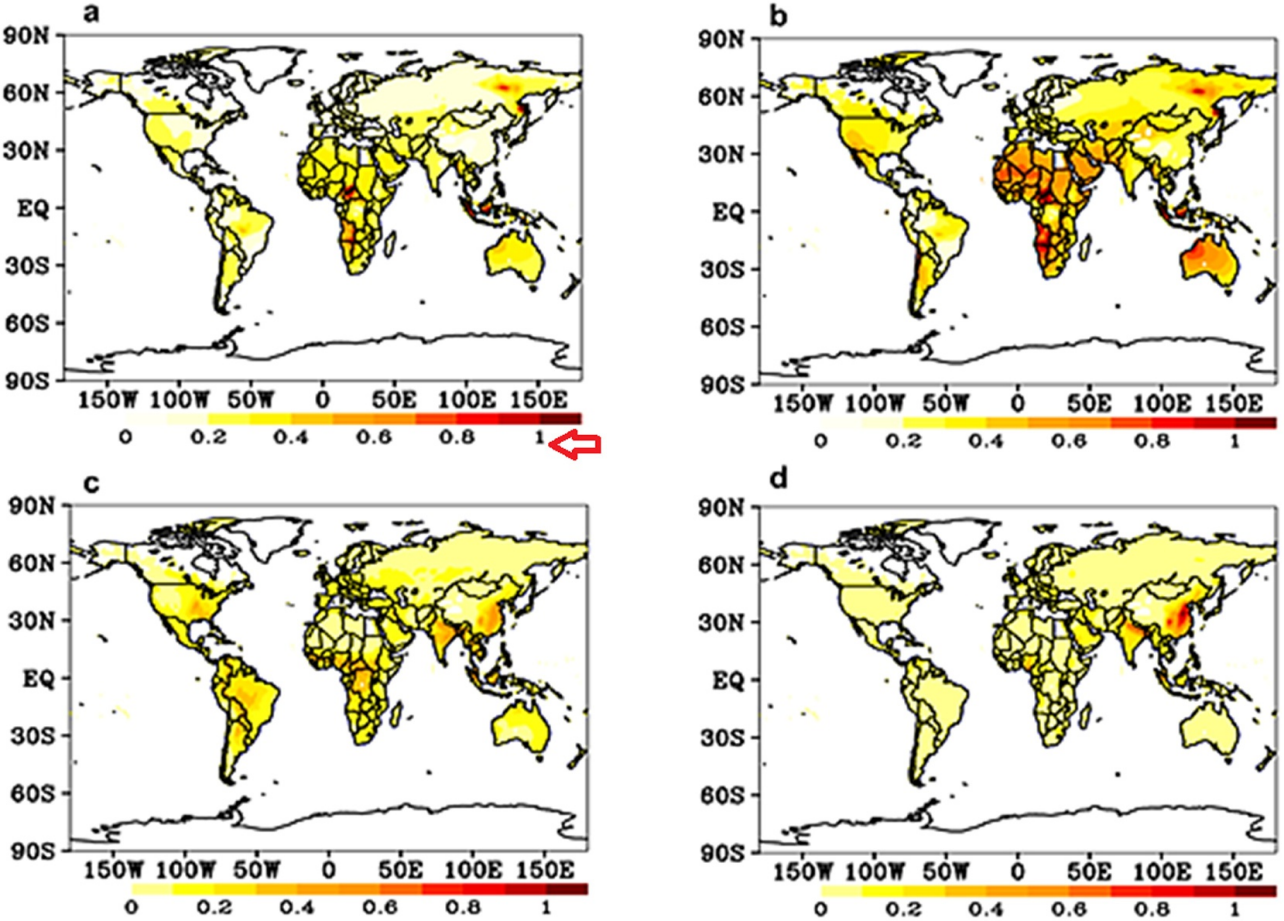
The chronic non‐cancer risks (mean HQ) associated with (a) Hydrogen cyanide, (b) Acetonitrile, (c) Formaldehyde and (d) Benzene from the baseline scenario. In the legend, a red arrow designates the acceptable limit of 1 for HQ.

Hydrogen cyanide exhibits moderate HQ values predominantly in the 0.2–0.6 range across most continental regions. Central Africa, parts of Southeast Asia, and tropical South America show elevated values approaching 1.0 (Figure 4a). However, no regions clearly exceed the critical threshold of HQ = 1, indicating relatively low health concern. Acetonitrile poses the greatest health risk among the four compounds, with a greater extent of regions with HQ values ranging from 0.4 to 1 (Figure 4b). Critical regions include central and eastern Africa (HQ > 1.0), parts of South and Southeast Asia, and portions of South America. Hydrogen cyanide and acetonitrile are primarily emitted from biogenic sources such as forests, marine environments, and volcanoes; consequently, these regions are strongly affected by hazardous biogenic VOCs. A recent study by (Duan et al., 2023) also suggests that South Asia and South America show an increasing trend in biogenic VOCs emissions (approx.10%).

Formaldehyde (Figure 4c) displays a globally distributed pattern with moderate HQ values of 0.4–0.8 across most inhabited continents. Central Africa and parts of Southeast Asia show the highest values approaching 0.8–1.0. Benzene shows a heterogeneous spatial pattern with distinct hotspots in South and Southeast Asia, where HQ values exceed 1.0, particularly over the Indian subcontinent (Indo‐Gangetic Plains) and parts of eastern China, as also suggested by Fan et al. (2021) for China and by Gautam et al. (2025) for India. Central Africa shows moderate to high HQ values (0.6–0.8), while Europe, North America, and Australia have lower HQ values (0.2–0.6). Benzene HQs exceeding 1 in densely populated South Asian regions pose an acute public health risk due to high population‐weighted exposures. A recent study by Padhi et al. (2025) reports that benzene and formaldehyde are key anthropogenic VOCs that dominate regions in East and South Asia, driven primarily by biomass burning and other anthropogenic activities. Therefore, based on the spatial extent and magnitude of HQ exceedances, the global relative risk of HazVOCs can be ranked as acetonitrile > benzene > formaldehyde ≈ hydrogen cyanide. Notably, acetonitrile exhibits the highest hazard quotient in our analysis; its atmospheric abundance is relatively low and largely associated with biomass burning (>90%), with fossil fuel sources contributing less than 6% to its global budget (∼0.8 Tg yr^−1^). In contrast, benzene has substantially higher emissions (∼24 Tg yr^−1^ from anthropogenic sources and 4–40 Tg yr^−1^ from biogenic sources (Table S5 in Supporting Information S1), while formaldehyde is linked to a much larger global NMVOC source (∼1,070 Tg yr^−1^) from vegetation, biomass burning, and anthropogenic activities. Thus, benzene and formaldehyde have greater atmospheric abundance and broader, more diverse source contributions (Table S5 in Supporting Information S1). We therefore focused on these compounds because they better represent widespread, multi‐source exposure across different regions.

#### 3.2.2 Regional Variability in Benzene and Formaldehyde Hazard Quotient

Figure 5 shows the regional non‐cancer Hazard (expressed as HQ) obtained for benzene and formaldehyde across scenarios. Across all regions, the DVOCS scenario increases HQ by 1.5–2x relative to the baseline, indicating strong sensitivity of population‐relevant exposure to increased VOCs emissions. In regions such as China and India, median HQ values approach or exceed unity (HQ ≈ 1.2–1.9), corresponding to a 21.5%–33% rise, implying a potential transition from low to moderate non‐cancer health concern under high‐emission conditions. In contrast, South Asia (S. Asia), Central Africa (CA), and South America (SAM) show comparatively lower mean increase in HQs (11%, 15%, and 7.6%, respectively). However, the upper quartiles under DVOCS still indicate localized hotspots of elevated risk.

**Figure 5:**
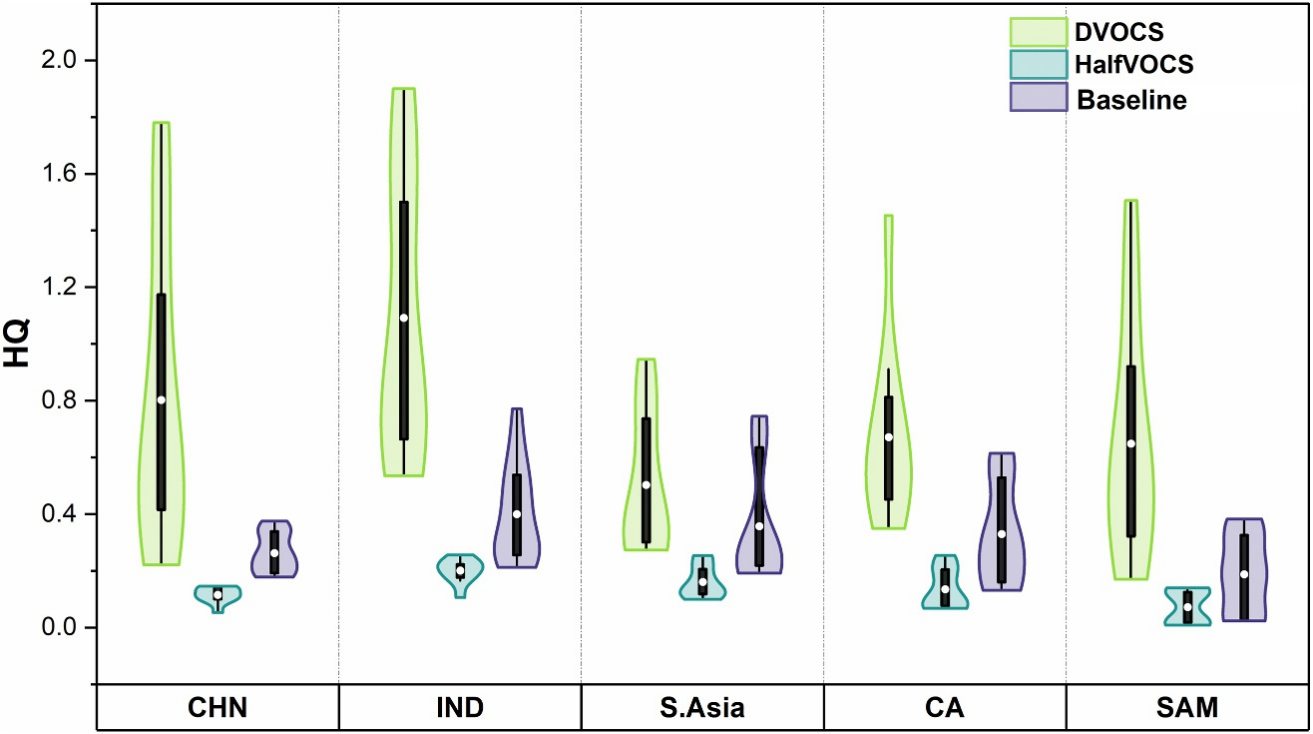
Chronic non‐cancer associated risks of benzene and formaldehyde under varying emission scenarios (DVOCS, HalfVOCS and Baseline).

The mean HQ values in the baseline scenario remain below unity in most regions (mean ≈ 0.25–0.8). Densely polluted regions such as India and China exhibit a larger increase in HQ in DVOCS than in the baseline scenario (18.4 ± 7.9% and 21.6 ± 11.7%, respectively). Under the HalfVOCS scenario, HQ values are reduced across regions by 36%–48% (mean‐43%) relative to the baseline scenario. In several regions, such as China, India, and Central Africa, the median HQs for HalfVOCs range from ≈0.1 to 0.2, suggesting that VOCs mitigation strategies can substantially reduce non‐cancer health risks associated with benzene and formaldehyde.

### 3.3 Population Exposure to Hazardous VOCs

Here we show the percentage change in population exposure for the DVOC and HalfVOC scenarios. Figures 6a and 6b quantify the sensitivity of population exposure to hazardous volatile organic compounds (HazVOCs), described in Tables S2 and S3 of Supporting Information S1; across five major global regions (India, China, South Asia, Central Africa, and South America). Under the DVOC scenario, all regions exhibit a robust increase in population exposure. The largest average increases are observed in China (21.6 ± 11.7%, 95% CI [14.3–29]) and India (18.4 ± 7.9%, 95% CI [13.5–23.3]), reflecting the combined influence of higher baseline anthropogenic emissions and population density. South Asia showed an increase of 10 ± 5.7%, whereas Central Africa and South America displayed comparatively moderate increases (8% and 6%, respectively). Notably, China and India, with pronounced deviations (wide interquartile ranges), are likely driven by regional emission source strengths and spatial population gradients. Statistical testing confirms that the exposure increases relative to baseline are significant in India (*p* = 0.0002), China (*p* = 0.00016), and South Asia (*p* = 0.02). At the same time, the response in Central Africa and South America is weaker but remains distinguishable from zero (*p* = 0.03 and 0.05).

**Figure 6:**
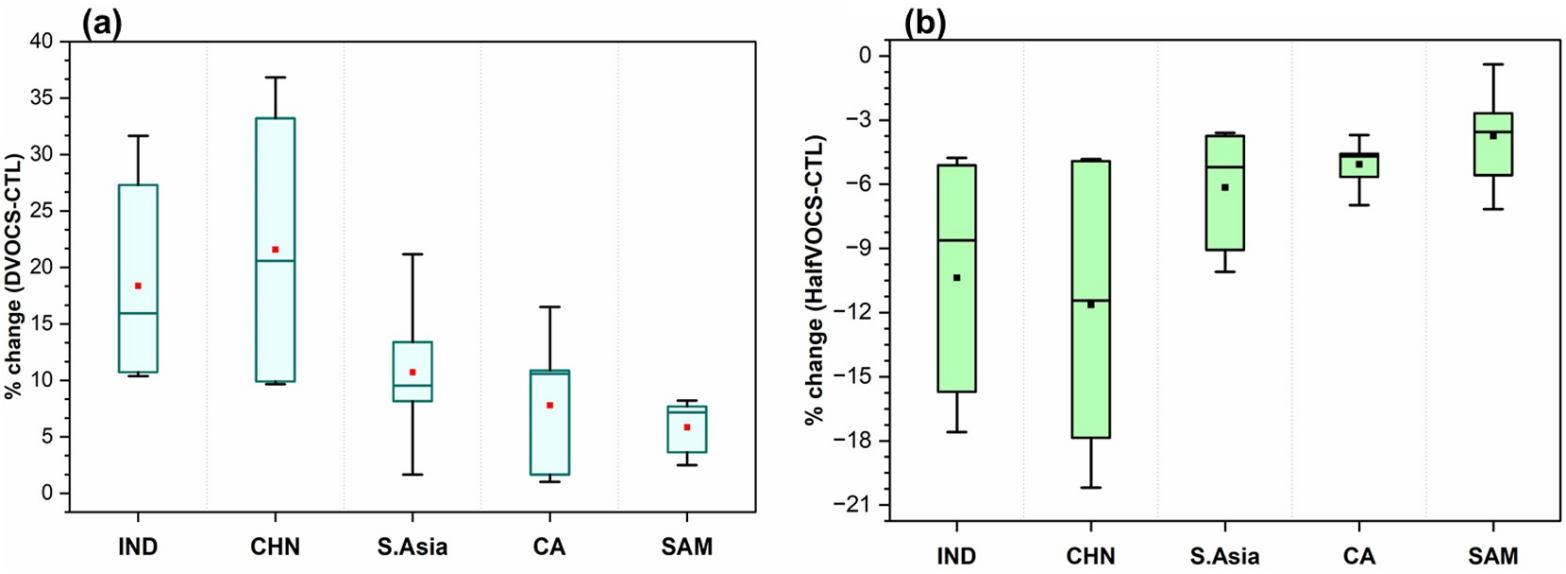
(a) Shows the percentage change in populations exposed to unacceptable HQ levels under DVOCS scenario and (b) is the same as (a) for the HalfVOCS scenario.

Conversely, VOCs reduction (HalfVOCS; Figure 5b) results in a consistent decrease in the exposed population across all regions, with all median values shifting below zero. China and India again show the strongest responses, with mean reductions of (−12 ± 6.4%, 95% CI [7.7–15.6]) and (10.4 ± 5.2%, 95% CI [6.8–13.7]), respectively. South Asia exhibited a moderate decrease (mean = −6.2%), whereas Central Africa and South America showed reductions of 5.5% and 4.2%, respectively. The IQR below zero across all regions indicate a statistically robust response, with reductions that are significant for India (*p* = 0.011), China (*p* = 0.0001), Central Africa (*p* = 0.02), and South Asia (*p* = 0.03). In summary, the global changes in the proportion of the exposed population across the enhancement and mitigation scenarios for HazVOCs indicate that higher concentrations were associated with markedly increased exposure.

### 3.4 Hazard Index

Figures 7a and 7b depicts the global distribution of the hazard index (HI) for respiratory and nervous system endpoints, calculated from the cumulative contribution of HazVOCs in the baseline scenario. Acute and chronic exposure to HazVOCs such as benzene, formaldehyde, acetonitrile, hydrogen cyanide, toluene, and xylene causes toxic effects on the human respiratory, nervous, reproductive, and endocrine systems (California EPA, 2015; Zhou et al., 2011). In cases of the respiratory and nervous systems, HI > 1 indicates major, undefined damage to these organs. However, the HIs < 1 for endocrine (HI = 0.4) and reproductive system (HI = 0.02) indicate no potential damage to these organs.

**Figure 7:**
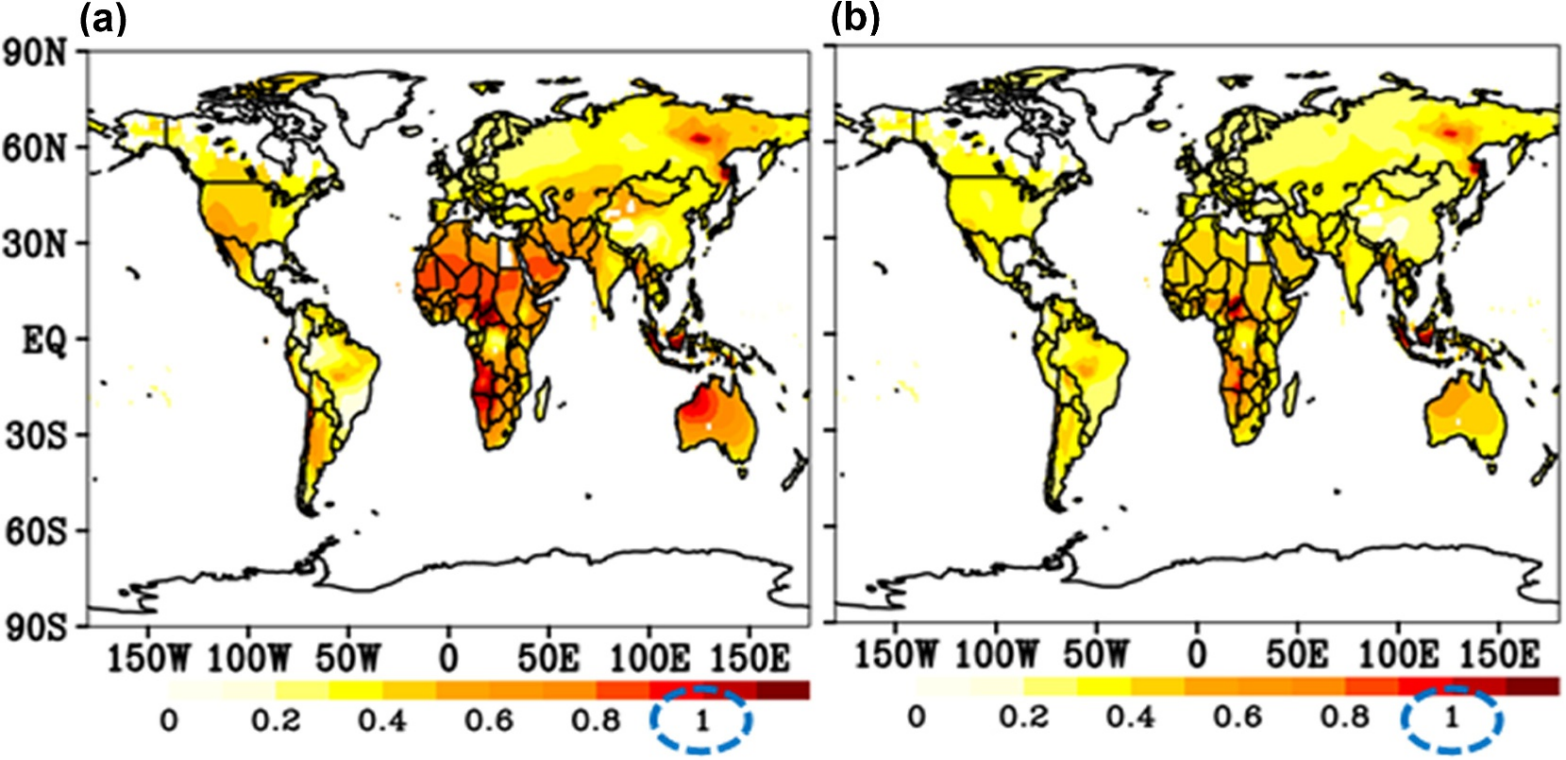
Non‐cancer associated risks w.r.t the two organ systems most susceptible to damage: (a) the respiratory system and (b) the nervous system, due to HazVOCs from 2001 to 2019.

Both organ systems (respiratory and nervous) exhibit coherent large‐scale spatial patterns, with higher HI values predominantly observed across large parts of Africa, South and East Asia, and parts of northwest Australia, indicating an increased potential for non‐carcinogenic health impacts. These regions are associated with intense anthropogenic activity, rapid urbanization, and substantial industrial and combustion‐related emissions (Huang et al., 2023).

In contrast, Figure 7 shows lower HI values confined to high‐latitude and remote regions with limited emission sources. These results underscore pronounced regional disparities in organ‐specific health risks and highlight the importance of targeted emission control strategies in regions with elevated cumulative VOC burdens.

## 4 Conclusion

We report the impact of Hazardous VOCs (HazVOCs) on HQ and HI hazard risk characterizing parameters using chemistry climate model simulations from three scenarios: (a) baseline scenario, (b) DVOCS and (c) Half VOCS. The baseline scenario reveals substantial, geographically heterogeneous non‐carcinogenic health risks from ambient VOCs exposure, requiring region‐specific mitigation strategies. The analysis reveals that acetonitrile, benzene, formaldehyde, and hydrogen cyanide exceed safe HQ thresholds primarily across South and Southeast Asia, with critical hotspots in eastern China, northern India, and Central Africa. The DVOCS scenario shows increases of 21%–33% in HQs in eastern China and the IGP region of India, and population exposure increases of 21.6 ± 11.7% and 18.0 ± 7.9%, respectively. It is larger than the other regions (5.6%–9.7%). Conversely, HalfVOCS reduces population exposure by approximately 11% in East and South Asia and 5.3% in other vulnerable regions. The average reduction across hotspots was 9.4 ± 2.8%. Above unity HI values were observed for the most vulnerable respiratory and nervous organ systems. Large parts of Africa, South and East Asia, and parts of northwestern Australia showed higher HI than the rest of the regions.

This dichotomy necessitates tailored interventions for industrial emission control in rapidly growing East Asian economies versus land‐use and biomass‐burning management in biogenic‐emission‐dominated regions. The findings provide an evidence base to strengthen VOC regulations where current standards are lenient, and to support their incorporation into routine air quality monitoring and governance frameworks where such provisions are currently lacking. By demonstrating the health benefits of VOC mitigation, this study supports more targeted regulatory actions to reduce exposure and protect population health at both regional and global scales.

## Conflict of Interest

The authors declare no conflicts of interest relevant to this study.

## Supporting information

Supporting Information S1

## Data Availability

Data on global population counts were obtained from the NASA Socioeconomic Data and Application Centre (SEDAC) for the Gridded Population of the World, version 4 (GPWv4) (https://data.nasa.gov/dataset/gridded‐population‐of‐the‐world‐version‐4‐gpwv4‐population‐count‐revision‐11). The model data and scripts used for health risk estimation and visualization in this article are available in Padhi et al. (2026, https://doi.org/10.5281/zenodo.19381465).
