# Supplementary material for: Global Health Hazards From Human‐Derived Volatile Organic Compounds Amid Emission Variability
Source: Geohealth. 2026 Oct 3;10(10):e2026GH001910. doi: 10.1029/2026GH001910 (PMC13633472; doi:10.1029/2026GH001910)
Supplement: Supplementary file 1 [file GH2-10-e2026GH001910-s001.pdf]

[*Geohealth*]

<u>Supporting Information for</u>

Global Health Hazard from Human-driven volatile organic compounds under emission variability

Annada Padhi, Suvarna Fadnavis and Mathew Koll Roxy

Centre for Climate Change Research, Indian Institute of Tropical Meteorology, Pune

India-411008

Contents of this file

Figure S1

Tables S1 to S5

Table S1 Model Configuration and simulation used in the study

Parameter	ECHAMHAM-MOZ Model configuration
Grid Dimension	$1^{\circ} \times 1^{\circ}$ 47 hybrid $\sigma - p$ levels
Vertical resolution	Surface to 0.01 hPa
Chemistry mechanisms	MOZ sub-model: describes the trace gas chemistry from the troposphere up to the lower thermosphere
	HAM sub-model: predicts the nucleation, growth, evolution and sinks of sulfate (SO_4), black carbon (BC), particulate organic matter (POM), sea salt (SS) and mineral dust (DU) aerosols
Simulation years	2001-2019
Emission inventory	The AEROCOM-ACCMIP-II emission inventory (Textor et al., 2006)
Experiment description	business-as-usual scenario; Increased emissions leading to doubling of anthropogenic VOCs-DVOCS; mitigation scenarios where emissions are reduced to half -HalfVOCS

Table S2 Toxicity criteria of selected HazVOCs in this study

S.No	HVOC Species	Reference exposure level (REL: μgm^{-3})
1	Formaldehyde (HCHO)	9
2	Acetaldehyde (CH3CHO)	140
3	Benzene (C6H6)	60
4	Methanol (CH3OH)	4000
5	Acetonitrile (CH3CN)	60
6	Hydrogen cyanide (HCN)	9
7	Methyl ethyl Ketone (MEK)	13000

8	Propylene (C_3H_6)	3000
9	Toluene (C_7H_8)	300
10	Xylene (C_8H_{10})	700
11	Carbon tetrachloride (CCl_4)	40

*All REL values obtained from the California EPA Risk Assessment Guidance Manual.

Table S3 REL values of VOCs used for Hazard Index (HI) of respective organ systems

Species Name	Acute Inhalation REL (μgm-3)	Acute Hazard Index Target Organ Systems
Acetaldehyde	4.7 x 10^2	Eyes; Respiratory System (sensory irritation)
	1.4 x 10^2	Respiratory System
Benzene	2.7 x 10^1	Reproductive/Developmental; Immune System; Hematologic System
	3.0 x 10^0	Hematologic System
Carbon Tetrachloride	1.9 x 10^3	Alimentary System (Liver); Nervous System Reproductive/Developmental
	4.0 x 10^1	Alimentary System (Liver) Development, Nervous System
Formaldehyde	5.5 x 10^1	Eyes (sensory irritation)
	9.0 x 10^0	Respiratory System
Hydrogen Cyanide	3.4 x 10^2	Nervous System

	9.0 x 10^0	Cardiovascular System; Endocrine System; Nervous System
Methanol	2.8 x 10^4	Nervous System
	4.0 x 10^3	Development
MEK	1.3 x 10^4	Eyes; Respiratory System
Xylenes	2.2 x 10^4	Eyes; Respiratory System; Nervous System
	7.0 x 10^2	Nervous System; Respiratory System, Eyes
Toluene	3.0 x 10^2	Development; Nervous System, Respiratory System
Acetonitrile	5.0 x 10^0	Respiratory System

#All values obtained from the California EPA Risk Assessment Guidance Manual

Table S4 Examples of VOC mitigation policies and regulatory measures by region

Region	Mitigation policies for VOCs	References
**European Union**	VOC Solvents Emissions Directive / solvent-use controls, emission limit values, reduction schemes, VOC paints directive	European Federation of Clean Air and Environmental Protection Associations (EFCA). (n.d.). EFCA. https://efca.net/?page_id=96
**United States**	EPA national VOC emission standards for consumer products under the Clean Air Act	U.S. Environmental Protection Agency. (2025). National volatile organic compound emission standards. https://www.epa.gov/stationary-sources-air-pollution/consumer-products-national-volatile-organic-compound-emission

**Canada**	Volatile Organic Compound Concentration Limits for Certain Products Regulations, product concentration limits for many product categories in Canada	Environment and Climate Change Canada.Volatile organic compounds in consumer and commercial products: Certain products fact sheet. https://www.canada.ca/en/environment-climate-change/services/managing-pollution/sources-industry/volatile-organic-compounds-consumer-commercial/certain-products/factsheet.html
**China**	VOC remediation plans, bans on high-VOC solvent-based coatings/inks/adhesives, and mandatory VOC content standards	SGS. (2020, August 19). China's Standardization Administration releases nine VOC standards. https://www.sgs.com/en/news/2020/08/safeguards-12420-chinas-standardization-administration-releases-nine-voc-standards

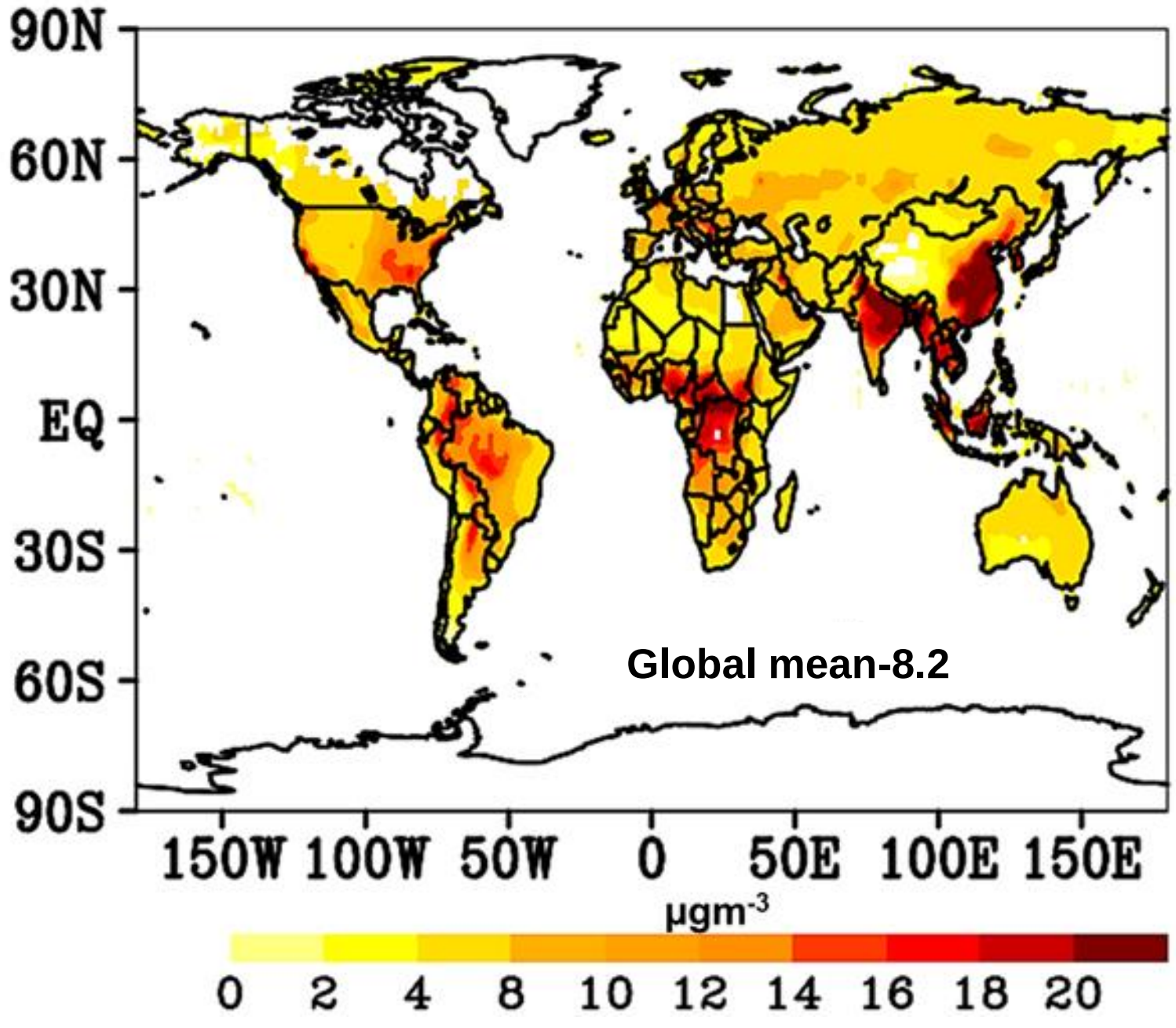

Figure S1 Global distribution of population-weighted concentration of total hazardous VOCs in 2001.

Supplementary Table S5. Comparison of global emission budgets for acetonitrile, benzene, and formaldehyde

Compound	Global Emission / Budget Estimate	Dominant Source	% from Single Dominant Source	Key Reference
**Acetonitrile (CH_3CN)**	~0.8 Tg yr^{-1} (total global budget)	Biomass burning	>90% [a]	Holzinger et al., 2001
**Benzene**	~24 Tg yr^{-1} (anthropogenic) + 4–40 Tg yr^{-1} (biogenic)	Mixed: vehicular, industrial, vegetative	No single dominant source	Misztal et al., 2015
**Formaldehyde (HCHO)**	~1070 Tg yr^{-1} precursor NMVOC pool (749 Tg vegetation + 102 Tg biomass burning + 219 Tg anthropogenic)	Secondary photochemical product [c]	Vegetation ~70% of precursor pool	Sfendla et al., 2026

[a] Fossil fuel combustion contributes an upper-limit estimate of ~0.021 Tg yr^{-1} (<6% of the total global CH_3CN budget); the remainder is attributed to biomass burning (Holzinger et al., 2001).

[b] Benzene emissions are distributed across vehicular exhaust, evaporative/industrial sources, and biogenic (vegetative) emission, with no single category dominating the global budget to the extent seen for acetonitrile.

[c] Formaldehyde is not primarily a directly emitted species; it is predominantly formed via atmospheric oxidation of a broad pool of precursor VOCs. The estimate shown therefore reflects the global precursor NMVOC flux rather than a direct HCHO emission inventory.

Note: Values represent point estimates or ranges as reported in the cited literature; formal uncertainty bounds were not available for all entries and are not included here.

References

California EPA.The air toxics hot spots program guidance manual for preparation of health risk assessments. (2015).

Holzinger, R., Jordan, A., Hansel, A., & Lindinger, W. (2001). Automobile Emissions of Acetonitrile: Assessment of its Contribution to the Global Source. *Journal of Atmospheric Chemistry*, 38, 187–193. https://doi.org/10.1023/A:1006435723375

Misztal, P. K., Owen, S. M., Guenther, A. B., et al. (2015). Atmospheric benzenoid emissions from plants rival those from fossil fuels. *Scientific Reports*, 5, 12064. https://doi.org/10.1038/srep12064

Sfendla, Y., Stavrakou, T., Müller, J.-F., Oomen, G.-M., Opacka, B., Danckaert, T., De Smedt, I., & Lerot, C. (2026). Global VOC emissions quantified from inversion of TROPOMI spaceborne formaldehyde and glyoxal data. *Atmospheric Chemistry and Physics*, *26*, 733–767. https://doi.org/10.5194/acp-26-733-2026

Textor, C., Schulz, M., Guibert, S., Kinne, S., Balkanski, Y., Bauer, S., Berntsen, T., Berglen, T., Boucher, O., Chin, M., Dentener, F., Diehl, T., Easter, R., Feichter, J., Fillmore, D., Ghan, S., Ginoux, P., Gong, S., Grini, A., Hendricks, J., Horowitz, L., Huang, P., Isaksen, I., Iversen, I., Kloster, S., Koch, D., Kirkevåg, A., Kristjansson, J. E., Krol, M., Lauer, A., Lamarque, J.-F., Liu, X., Montanaro, V., Myhre, G., Penner, J., Pitari, G., Reddy, S., Seland, Ø., Stier, P., Takemura, T., & Tie, X. (2006). *Analysis and quantification of the diversities of aerosol life cycles within AeroCom*. Atmospheric Chemistry and Physics, 6, 1777–1813. https://doi.org/10.5194/acp-6-1777-2006.
